# Fabrication and Property Characterization of Long-Glass-Fiber-Reinforced Polypropylene Composites Processed Using a Three-Barrel Injection Molding Machine

**DOI:** 10.3390/polym14061251

**Published:** 2022-03-20

**Authors:** Guan-Yan Liou, Chi-Wei Su, Po-Wei Huang, Sheng-Jye Hwang, Chao-Tsai Huang, Hsin-Shu Peng

**Affiliations:** 1 Department of Mechanical Engineering, National Cheng Kung University, Tainan 70101, Taiwan; n16094182@gs.ncku.edu.tw (G.-Y.L.); hyde00155@gmail.com (C.-W.S.); 2 Department of Mechanical and Computer-Aided Engineering, Feng Chia University, Taichung 40724, Taiwan; bowei8915@gmail.com (P.-W.H.); hspeng@fcu.edu.tw (H.-S.P.); 3 Department of Chemical and Materials Engineering, Tamkang University, New Taipei 251301, Taiwan; cthuang@mail.tku.edu.tw

**Keywords:** long-fiber-reinforced thermoplastic, fiber length, fiber orientation, injection molding, mechanical properties

## Abstract

Processing equipment and parameters will highly influence the properties of long-fiber-reinforced injection-molded thermoplastic composites, leading to different fiber lengths and orientations. Thus, maintaining fiber length during the injection molding process is always a big challenge for engineers. This study uses long-glass-fiber-reinforced polypropylene with 25 mm fiber length and a special-built novel injection molding machine with a three-barrel injection unit, including a plasticizing screw, an injection plunger, and a packing plunger, to fabricate injection molding parts while retaining long fiber length. This study also discusses the influence of process parameters, such as back pressure, screw speed, melt temperature, and different flow paths, on the properties of long-glass-fiber-reinforced composites. The experiment results show that a higher screw speed and back pressure will reduce the fiber length in the injection-molded parts. However, using appropriate parameter settings can maintain the fiber length to more than 10 mm. It was found that by increasing the back pressure, the cross direction of the fiber orientation can be increased by up to 15% and the air trap volume fraction can be decreased by up to 86%. Setting appropriate back pressure under a low screw speed will increase the tensile strength. Finally, it was found that the single-edge-gate path results in a higher tensile strength than that of the single-sprue-gate path due to the retainment of longer fiber length in the injection-molded part.

## 1. Introduction

The automotive industry has used many lightweight composite materials to reduce weight and improve fuel efficiency in recent years. Among them, fiber-reinforced thermoplastic composites are popular in the automotive industry. Fiber-reinforced composites have the characteristic of lightweight and can effectively improve the mechanical properties of the product [1,2,3]. Automobile components widely use plastic injection molding products. However, injection molding processing methods will change the fiber characteristics (fiber length, fiber orientation, and fiber weight fraction) and affect its mechanical properties. Therefore, the literature collected below studies the influence of fiber characteristics, the injection molding machine, mold design, and injection parameters on the mechanical properties of the fiber.

Fiber characteristics significantly affect the mechanical properties of the product. Thomason [4] and Sun et al. [5] mentioned that the mechanical properties of long-fiber-reinforced thermoplastics depend on three main factors, which are fiber length, fiber orientation, and fiber weight fraction. Baillif and Oksman [6] found that both fiber length and fiber orientation affect mechanical properties but that the effect of fiber length is more significant. In the injection molding process, the melt flow process affects the degree of fiber orientation. Advani and Sozer [7] mentioned that injection-molded fiber-reinforced thermoplastic parts typically have a skin/shell/core structure. Jan et al. [8] mentioned that the degree of fiber alignment in the flow direction is sensitive to fiber weight fraction. Toll and Anderson [9] found that the core regions of samples made with long-fiber compounds have a higher fiber content and a more consistent fiber orientation along the flow direction than those made with short-fiber compounds. Wang et al. [10] showed that the fiber orientation in the loading direction is beneficial for improving the tensile strength and modulus of long-fiber-reinforced thermoplastic composites. Kumar et al. [11] noted that as the fiber content increases, the tensile strength and impact resistance of the molded parts also increase. Therefore, maintaining fiber integrity and achieving dense and uniform fiber orientation in the final product are critical challenges for injection molding.

The gate, the runner, the cavity design, and the injection molding machine are also essential. The flow path design of the composite material melt can damage the fiber. Hung and Tseng [12] pointed out that the threads of long-fiber-reinforced thermoplastic materials break when they move through the plasticizing screw, so it is not easy to use injection molding processing to inject long-fiber-reinforced thermoplastics. Güllü et al. [13] found that increasing the cross section of the gate can help produce a more extended fiber distribution and increase the tensile strength. Lafrance and Krawczak [14] showed that the quality of the product depends on the gate type and the design of the plasticizing unit. In addition, the gate location and size affect the composite’s skin/shell/core structure during the filling stage. Huang et al. [15] investigated the effects of the process parameters on the fiber length and tensile strength of long-fiber-reinforced thermoplastic parts produced using a customized injection molding machine with a novel three-barrel-type injection unit. The results showed that the fibers in the molded part of the unit maintain an average length of more than 13 mm, given appropriate settings of the process parameters. According to the result of the references taken into consideration, this study uses an injection molding machine with a three-barrel injection molding unit, including a plasticizing screw, an injection plunger, and a packing plunger. This design of the injection unit can effectively avoid damaging the fiber and maintain the fiber length during the injection molding process.

The injection parameter setting is also a crucial part. Improper processing parameter setting may cause defects, such as fibers and bubbles, further reducing the product’s mechanical properties. Some studies have pointed out the influence of injection molding process parameters on long-fiber-reinforced thermoplastic products. According to Sanou et al. [16], the melt temperature and the fiber content significantly affect the fiber orientation. Lafrance and Krawczak [17] mentioned that the most critical factors affecting bending strength and modulus are plasticizing, filling, and post-filling stages (melt temperature, volume flow, back pressure, and screw speed). Salleh et al. [18] found out that high-temperature processing can increase the tensile modulus of composite materials but the performance will decrease during low-temperature processing, especially under a high fiber content. Yilmazer and Cansever [19] showed that the average fiber length decreases when the shear rate increases on changing the screw speed or the feed rate. The impact strength, the tensile modulus, and the tensile strength increase, while the elongation at break decreases as the average fiber length increases. Rohde et al. [20] showed that backpressure harms fiber length and affects the energy absorption of long-fiber-reinforced thermoplastic parts. Hagstrand et al. [21] and Peng [22] mentioned that the existence of air traps in fiber-reinforced composites significantly damages their mechanical performance. According to the above literature review, the melt temperature, the back pressure, and the screw speed are important manufacturing parameters that affect the characteristics and mechanical properties of the fiber.

However, how to catch those fiber microstructures in the final parts is still challenging. In general, two methods have been used. One is the optical microscopy method, and the other is the micro-computerized tomography scan method. Bernasconi [23] mentioned that the optical and micro-computed methods can provide similar results in fiber orientation. Huang et al. [24] mentioned the fiber orientation distribution using micro-computerized tomography scan and image analysis by Avizo software. The correlation between the fiber microstructure feature (particularly in fiber orientation) and tensile modulus and tensile stress for fiber-reinforced thermoplastic in the injection molding process can be validated. Since the fiber length of the samples used in this study is extremely long, a big volume of a sample part needed to be examined in order to calculate the fiber length and orientation in the sample. A high-resolution computerized tomography scan is not practical since it requires too many slices of a big cross section and the generated data size exceeds the handling capability of a modern computer. Therefore, this study uses the optical microscopy method and image analysis by Avizo software to examine the fiber microstructures.

Based on the literature collected above, the present study uses the three-barrel injection molding machine to identify the effects of the injection molding process parameters (namely, the backpressure, the screw speed, and the melt temperature) and two different melt flow paths (single-edge gate and single-sprue gate) on four fiber characteristics of molded long-fiber-reinforced thermoplastic parts: the fiber length, the fiber orientation, the fiber weight fraction, and the air trap. Finally, the tensile test for the long-fiber-reinforced thermoplastic samples is conducted. To validate the optical microscopy scan method in this study, the results are compared to the fiber length and fiber orientation measurement results using high-temperature sintering and computed tomography. The images of the optical microscopy scan and computed tomography are imported to Avizo software for fiber orientation analysis results. The fiber orientation defined by Advani and Tucker [25] presents a model based on even-order tensors to describe the average orientation of the fibers. The results showed that in terms of describing the fiber orientation distribution through the part thickness direction, the tensor components a11, a22, and a33 were sufficient for physical interpretation purposes, where axis 1 was aligned with the flow direction, axis 2 was aligned with the cross flow direction, and axis 3 was aligned with the thickness direction.

## 2. Materials and Methods

### 2.1. Material Fabrication

**Figure 1:**
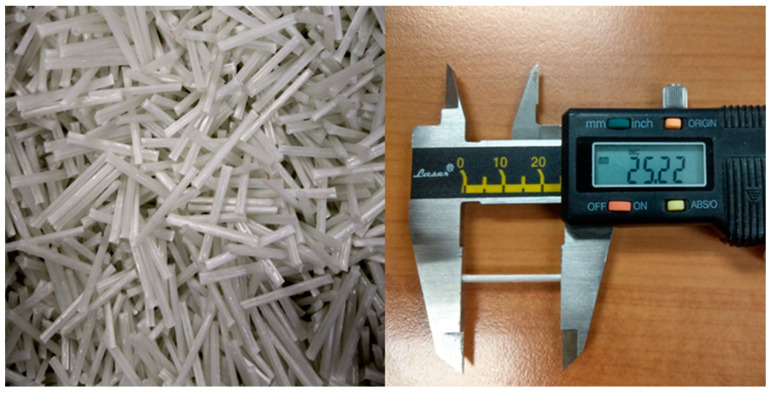
Long-glass-fiber pellets.

Long-glass-fiber-reinforced polypropylene was chosen for the experiment. LGP503NA250, which has a fiber content of 50 wt.%, was used as the molding material for the experiment. The length of the sample was 25 mm, and the diameter was 17 μm (as shown in Figure 1). The material was supplied by GECO (Great Eastern Resins Industrial Co. Ltd., Taichung, Taiwan).

### 2.2. Injection Molding Machine

**Figure 2:**
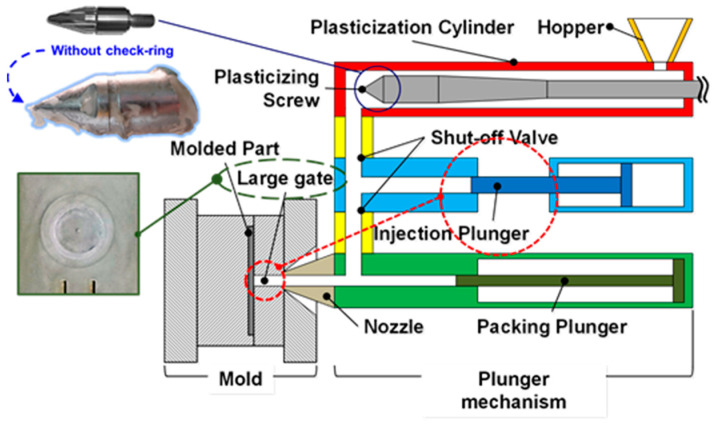
Design and application of the injection molding machine using a three-barrel injection mechanism (plasticization stage, filling stage, and packing stage).

A 180-ton injection molding machine was used for the experiment (CLF, Chuan Lih Fa Co., Ltd., Tainan, Taiwan) at a maximum injection speed of 150 mm/sec and a maximum injection pressure of 177 MPa. In an attempt to maintain the initial fiber length during the injection molding process, a three-barrel injection unit was designed and developed by the researchers (Figure 2). The injection unit consisted of a plasticization screw (φ = 55 mm), an injection plunger (φ = 45 mm), and a packing plunger (φ = 40 mm). The plasticization screw used in the injection unit was designed to have a low shear strain rate by reducing the compression ratio of the screw to minimize the fiber breakage rate. The screw does not move back and forth, so it does not have a check ring. The injection and packing plungers store and deliver composite melt into the mold cavity. This design of the injection unit can effectively avoid damaging the fiber and maintain the fiber length during the injection molding process. A cooling channel is designed and built in the packing plunger of the novel injection molding machine so the process cycle time can be almost the same as that of the traditional injection molding process, even though the gate size of the mold is considerably big.

### 2.3. Sample and Mold

**Figure 3:**
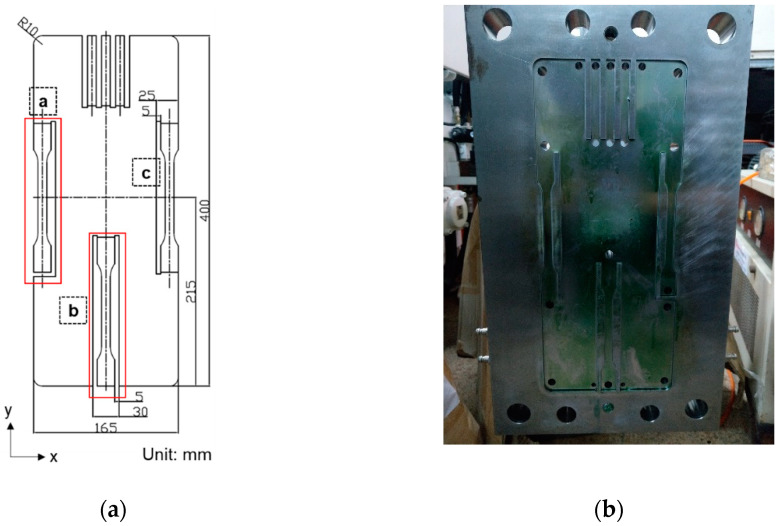
Schematic of tensile samples: (**a**) diagram of the sample designs (ISO 527-2 at a thickness of 3 mm) and (**b**) diagram of the mold cavity of the samples.

To investigate the effects of the melt flow path on the mechanical properties of the long-fiber-reinforced thermoplastic components, a mold was designed with three different flow paths, (a) a single-edge gate, (b) a single-sprue gate, and (c) a double-edge gate, as shown in Figure 3 (Note that in designing the mold, the double-edge-gate path is intended to allow observation of the effects of the process parameters on the weld line. However, this issue is out of the scope of the present study, and the double-edge-gate path is, therefore, ignored in the remaining discussion).

### 2.4. Optical Microscopy Scan Method

**Figure 4:**
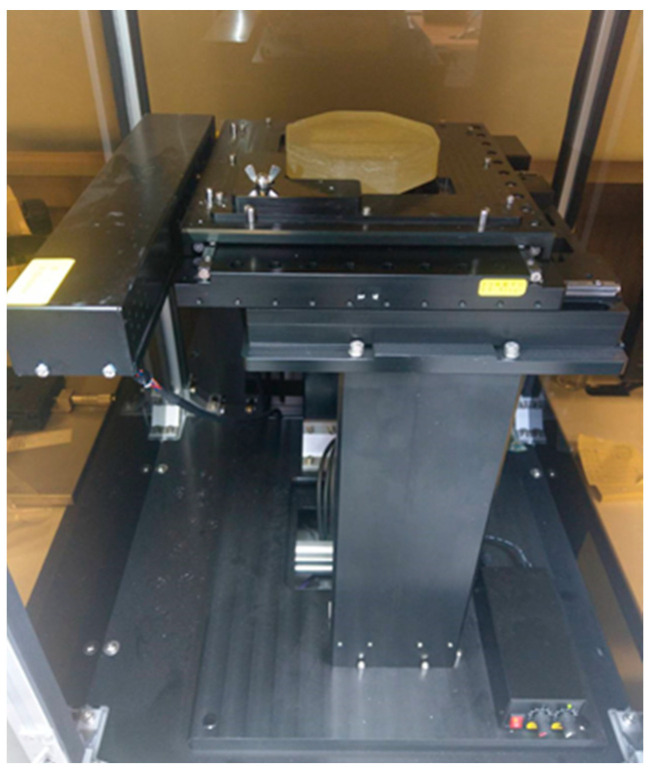
Optical microscopy scanner.

The optical microscope scanner (shown in Figure 4) was developed with a PC-based control system with a 10× magnification lens resolution of 2 μm. The typical single scanned picture was 0.9 mm × 0.9 mm in size. The camera took some pictures in a large area by controlling the X and Y axes using a computer and then stitched them into a single image. The optical microscopy method was carried out by grinding and polishing the sample, after which, it was placed on the platform of the optical microscope to obtain a surface image. After the scanning process, we continued grinding and polishing (0.2 mm in depth). The entire process was repeated iteratively until sample images were acquired from the skin layer to the core layer. Finally, the images obtained using the optical microscopy method were imported to Avizo for analysis.

### 2.5. Image Analysis Software

Avizo (version 9.7.0, Thermo Fisher Scientific, Waltham, MA, USA) is a software program that enables users to perform interactive visualization and computation on 3D data sets. In the present study, the built-in fiber tracing module and the built-in thresholding module functions were used to analyze the fiber length, orientation, and weight fraction and the volume fraction of air the traps.

### 2.6. Experimental Parameters

The injection molding experiments considered the effects of three different process parameters on the fiber characteristics and mechanical properties of the long-glass-fiber-reinforced thermoplastic parts of interest, the back pressure (0–1.12 MPa), the plasticization screw speed (60–150 rpm), and the melt temperature (230–250 °C), as shown in Table 1. To validate the optical microscopy results for the fiber length and fiber orientation, high-temperature sintering and computed tomography, respectively, were used under the same processing conditions. A back pressure of 0.7 MPa, a screw speed of 90 rpm, and a melt temperature of 230 °C were used.

### 2.7. Characterization of the Molded Parts

#### 2.7.1. Fiber Length

**Figure 5:**
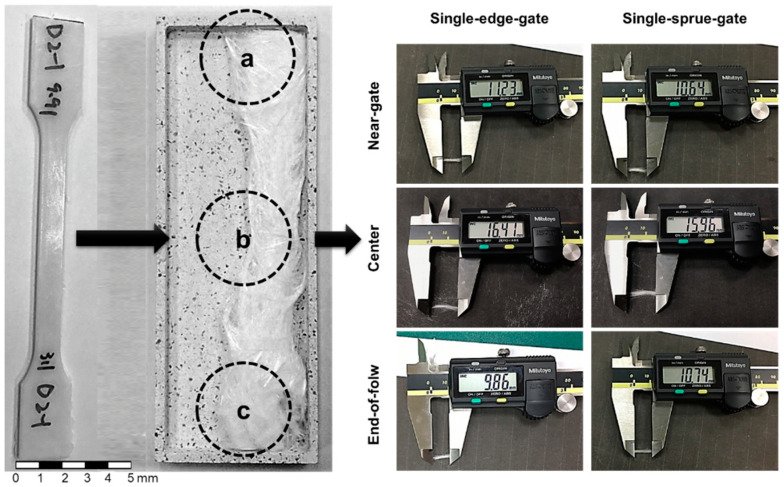
Measurement of the fiber length after high-temperature sintering for different melt flow paths: (**a**) the near-gate region, (**b**) the center region, and (**c**) the end-of-flow region.

To validate the feasibility of the optical microscopy method, the fiber length results after high-temperature sintering and optical microscopy scanning were compared. High-temperature sintering was carried out exposing the sample to a high temperature to make the polypropylene melt and leave only the glass fiber. The fiber length was obtained by measuring and averaging 200 fibers (shown in Figure 5). The fiber length was analyzed in this study by importing images from the optical microscopy scans into Avizo’s built-in fiber-tracking module.

#### 2.7.2. Fiber Orientation

**Figure 6:**
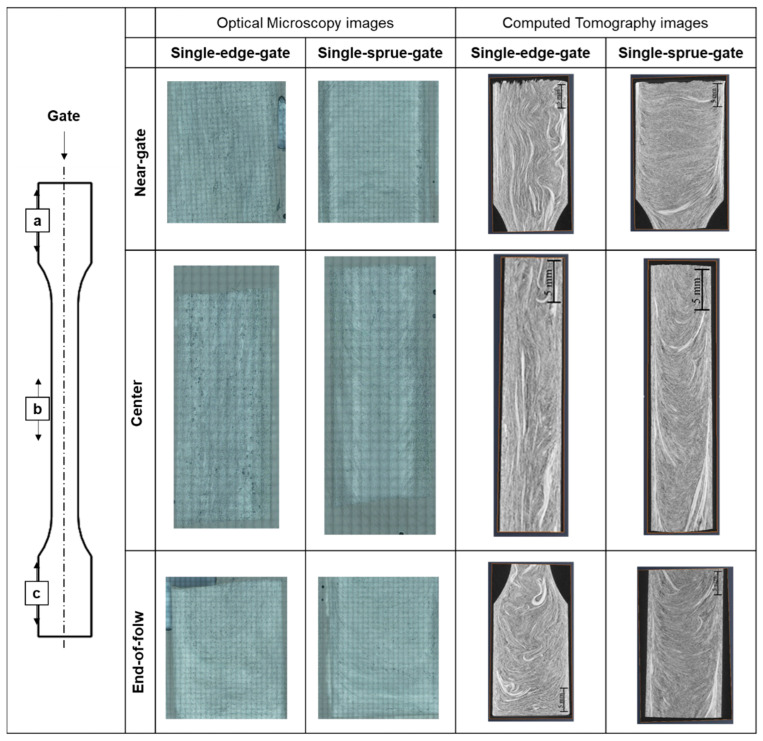
Observation of optical microscopy and computed tomography images (core layer) for different melt flow paths: (**a**) the near-gate region, (**b**) the center region, and (**c**) the end-of-flow region.

To validate the feasibility of the optical microscopy method, the fiber orientation results for the computerized tomography and the optical microscopy methods were compared. The computerized tomography scan was performed using the Zeiss Metrotom 800 scanner (Carl Zeiss AG, Jena, Germany). Table 2 shows the computerized tomography parameters. Since the fiber length of the samples used in this study can be extremely long, a big volume of a sample part needed to be examined in order to calculate the fiber length and orientation in the sample. A high-resolution computerized tomography scan is not practical since it requires too many slices of a big cross section and the generated data size exceeds the handling capability of a modern computer. The authors are perfectly aware of possible problems caused by not using a fine slicing computerized tomography scan to determine fiber length and orientation. Thus, this study not only used not-that-fine computerized tomography scan but also used an optical microscopy scan approach and high-temperature sintering to examine the fiber characteristics (fiber length and fiber orientation) in order to ensure the correctness of the analysis. The analysis of the fiber orientation was accomplished by importing images from computed tomography and optical microscopy into Avizo’s built-in fiber-tracking module. Figure 6 shows the core layer images obtained using computed tomography and optical microscopy.

The fiber orientation was examined using the model proposed by Advani and Tucker [10]. In theory, the second-order tensor represents a fiber orientation that is applied to rigid fibers only. Hence, strictly speaking, it cannot be used for the injection-molded long-fiber-reinforced thermoplastic composites considered in the present study since the fibers typically bend during the molding process and, therefore, do not always have a rigid rod shape. Nonetheless, the results still provide a useful qualitative description of the fiber orientation and were thus still retained in the present study.

#### 2.7.3. Fiber Weight Fraction

The glass fibers appeared as white stripes in the optical microscopy images in the model and were detected automatically by setting appropriate threshold values for the grayscales of the scanned images in Avizo. The fiber fraction volume could then be determined by computing the data detected in white regions using Avizo’s built-in thresholding modules. The fiber volume fraction within the various samples was determined by setting thresholding values for the grayscales and coloring the white-striped regions in the images in the Avizo model. Different from determining the air trap content, the white-striped regions in the images represented the glass fibers that could be detected and calculated using the built-in modules. The fiber weight fraction was then computed using polypropylene and glass fiber densities of 0.90 g/cm^3^ and 2.54 g/cm^3^, respectively.

#### 2.7.4. Air Trap Volume Fraction

The air trapped within the sample appeared as black regions in the model and was detected automatically by setting appropriate threshold values for the grayscales of the scanned images in Avizo. The air trap volume fraction could then be determined by computing the data detected in the black regions using the built-in thresholding modules.

### 2.8. Tensile Test

In accordance with the ISO 527-2 standard, the test specimens were designed with a length of 170 mm, a width of 10 mm, and a thickness of 3 mm. The tensile tests were conducted on an AG-100 KN machine (Shimadzu Scientific Instruments Co. Ltd., Taipei, Taiwan) with a maximum load of 1000 kgf and a constant crosshead speed of 20 mm/min. For each set of experimental conditions and for both flow paths (i.e., the single-sprue gate and the single-edge gate), 20 tensile tests were performed, with the results averaged to obtain a final representative tensile strength value.

## 3. Results and Discussion

### 3.1. Comparison of High-Temperature Sintering and the Optical Microscopy Method

**Figure 7:**
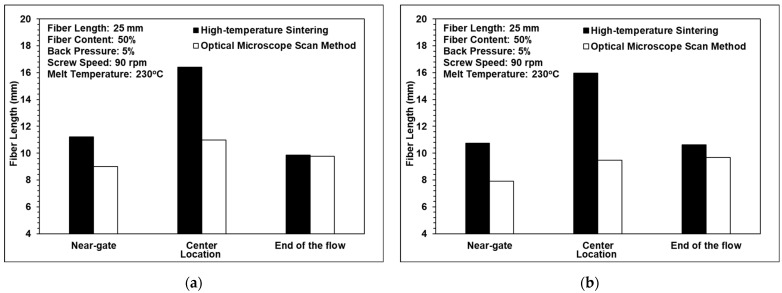
Comparison of fiber lengths using high-temperature sintering and optical microscopy for (**a**) the single-edge gate and (**b**) the single-sprue gate.

Figure 7 compares the fiber length results obtained from the high-temperature sintering and the optical microscopy images. It can be observed that the sample lengths after high-temperature sintering were longer than those measured using optical microscopy in each of the scanning locations. The difference between the two results is attributed to the greater interval (0.2 mm) between the sequential layers in the images obtained using optical microscopy. Consequently, fibers may be erroneously detected as discontinuous segments if they pass through multiple layers of the Avizo model. However, despite the difference in the absolute values of the fiber length, the two methods showed a similar trend in terms of the variations in the fiber length in different regions of the sample. The study shows that the central region of the sample has the longest fiber length. However, for the single-sprue-gate part, the optical microscopy result shows that the longest fiber length is at the end of the mold cavity. According to the results, the fiber lengths at the two locations are considerably close. Thus, the trend that the central region of the sample has the longest fiber length can be justified.

### 3.2. Comparison of the Computerized Tomography and Optical Microscopy Results

**Figure 8:**
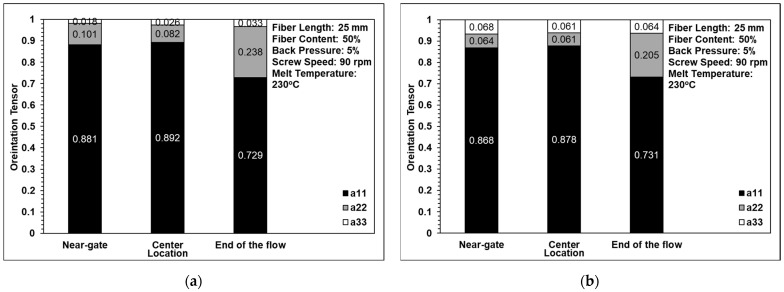
Comparison of orientation tensor in (**a**) computed tomography and (**b**) optical microscopy images of a single-edge gate.

**Figure 9:**
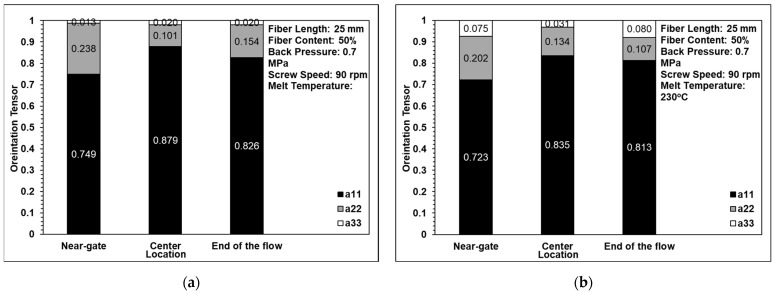
Comparison of the orientation tensor in (**a**) computed tomography and (**b**) optical microscopy images of a single-sprue gate.

Figure 8 and Figure 9 show the computed tomography and optical microscopy results for the fiber orientation within the single-edge-gate and simple-sprue-gate samples, respectively. Note that, as described in Section 1, notations a11, a22, and a33 refer to the flow direction, the cross flow direction, and the thickness direction, respectively. For both sample types, good qualitative agreement existed between the computed tomography image and each region of the sample. The number of fibers aligned in the flow direction was slightly higher in the computed tomography images than in the optical microscopy images since, in the latter case, the fibers may have been falsely identified as discontinuous segments due to the larger spacing distance between adjacent layers. For both models, and both sample types, the maximum number of fibers aligned in the flow direction in the central region due to the contraction of the specimen geometry, which constrains the flow in an axial direction [22]. In contrast, there were a11 tensor component reductions in the end-of-flow region due to the expansion of the specimen structure, which allowed freer flow in multiple directions as it filled the mold.

### 3.3. Fiber Length Analysis

**Figure 10:**
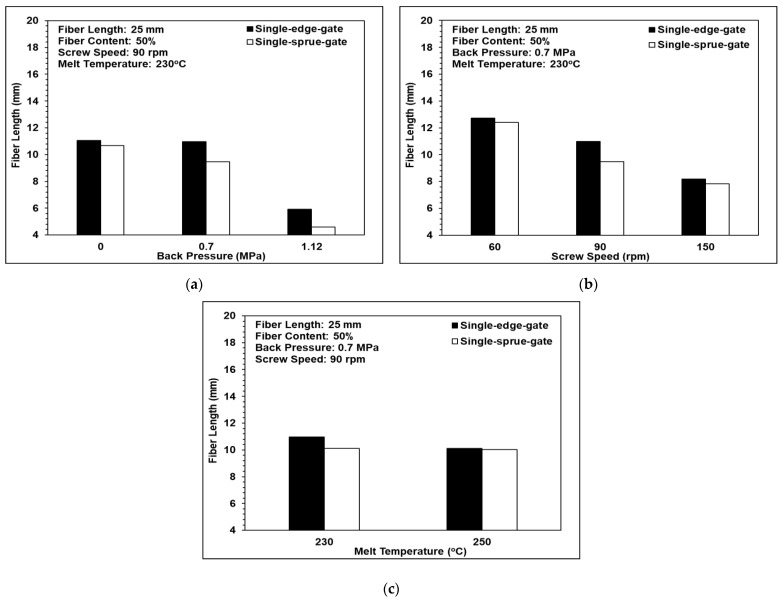
Effects of the different melt flow paths on fiber length: (**a**) back pressure variations, (**b**) screw speed variations, and (**c**) melt temperature variations.

Figure 10 shows the effects of the back pressure, the screw speed, and the melt temperature on the fiber length. It can be observed that a fiber length of approximately 10 mm was maintained when the back pressure and the screw speed were assigned low values, of 0.7 MPa and 90 rpm, respectively. In other words, the three-barrel injection molding unit successfully protects the fibers from serious damage during the molding process provided that the processing conditions (back pressure and screw speed) are appropriately set. As the back pressure and the screw speed increase, the shear strain rate acting on the polymer melt also increases and, hence, the fibers tend to break. However, as shown in Figure 10c, the melt temperature had almost no effect on the fiber length. The result is similar to that of the previous research by Huang et al. [26].

For all three processing conditions, the fiber length in the central region of the single-edge-gate samples was longer than that in the single-sprue-gate samples. This finding is reasonable since the flow distance for the single-edge-gate cavity from the injection nozzle was longer than that for the single-sprue-gate cavity. As a result, the pressure gradient and the shear strain rate were both reduced and, hence, the fiber underwent less deformation and damage during the molding process.

### 3.4. Fiber Orientation Analysis

**Figure 11:**
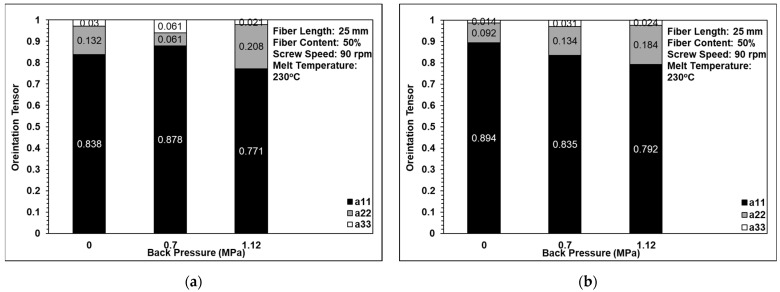
Effects of back pressure on fiber orientation in (**a**) the single-edge gate and (**b**) the single-sprue gate.

**Figure 12:**
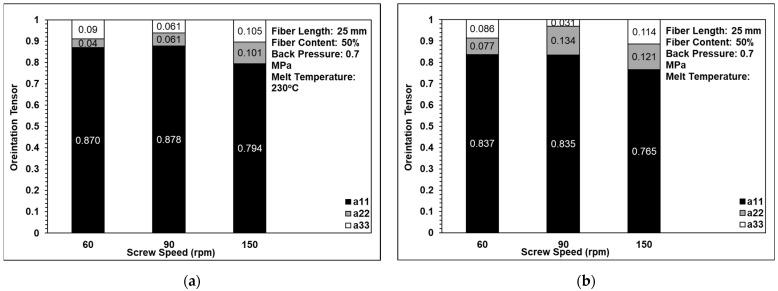
Effects of screw speed on fiber orientation in (**a**) the single-edge gate and (**b**) the single-sprue gate.

**Figure 13:**
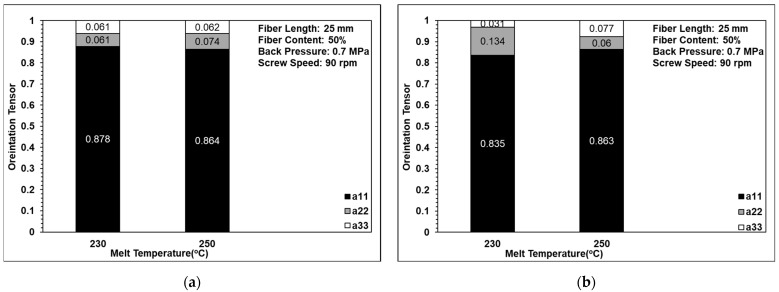
Effects of melt temperature on fiber orientation in (**a**) the single-edge gate and (**b**) the single-sprue gate.

Figure 11 shows the effects of back pressure on the fiber orientation in the single-edge-gate and single-sprue-gate samples. Note that, as described in Section 1, notations a11, a22, and a33 refer to the flow direction, the cross flow direction, and the thickness direction, respectively. Figure 12 shows the equivalent results for the effects of screw speed, and Figure 13 shows the effects of the melt temperature. In general, the results indicate that, for all of the considered processing conditions, most of the fibers were aligned in the flow direction due to the shallow geometry of the tensile specimens and the long-fiber nature of the composite material. The number of fibers oriented perpendicularly to the flow direction increased slightly as the back pressure and the screw speed were increased since more severe fiber breakage leads to shorter fibers under such conditions. For example, when the back pressure values are set to 0, 0.7, and 1.12 MPa, the cross directions of the fiber orientation are 0.132, 0.016, and 0.208, respectively. This means that when the back pressure increased, the cross-direction fiber orientation of the single-edge gate and the single-sprue gate increased to 15% and 9%, respectively. When increasing the screw speed, the cross direction of the single-edge gate and the single-sprue gate increased to 4% and 8%, respectively. However, the melt temperature had little effect on the fiber orientation. The melt flow path also had only a minor effect on the orientation of the fibers in the present study. When increasing the melt temperature, the cross-direction fiber orientation of the single-edge gate and the single-sprue gate changed to 1% and 7%, respectively. Sanou et al. [16] mentioned that the melt temperature affects the fiber orientation in the short-fiber-length case. However, in this study, the fiber length is considerably long, so the injection parameters will have only a small effect on the fiber orientation.

### 3.5. Fiber Weight Fraction Analysis

**Figure 14:**
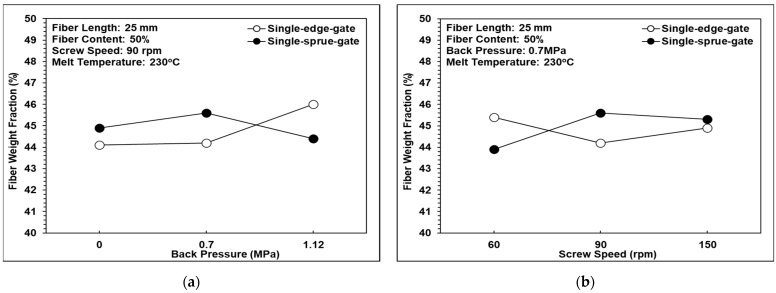

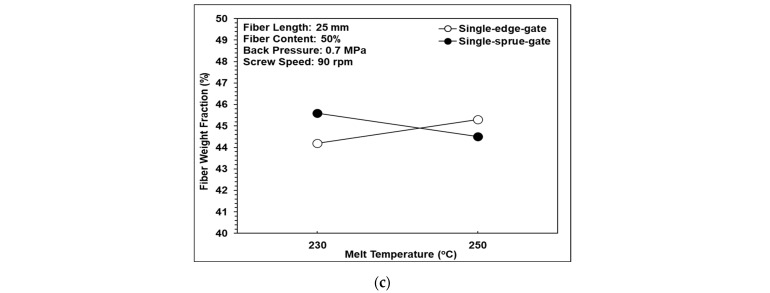
Effects of the different melt flow paths on the fiber weight fraction: (**a**) back pressure variations, (**b**) screw speed variations, and (**c**) melt temperature variations.

Figure 14 shows the effects of back pressure, screw speed, and melt temperature, respectively, on the fiber weight fraction. In general, the results indicate that neither the process parameters nor the melt flow path significantly affect the weight fraction of the fibers. This study is more focused on the influence of fiber length and orientation on the properties of the injection-molded long-fiber-reinforced parts, and the fiber weight fraction analysis is just used to validate that the fiber content is close to 50 wt.%. According to the analysis results, the trends in the fiber weight fraction tend to be constant and do not change significantly under different process conditions. For example, the weight fraction of the single-sprue gate at 60, 90, and 150 rpm of screw speed resulted in 44, 46, and 45 wt.%, respectively.

### 3.6. Air Trap Volume Fraction Analysis

**Figure 15:**
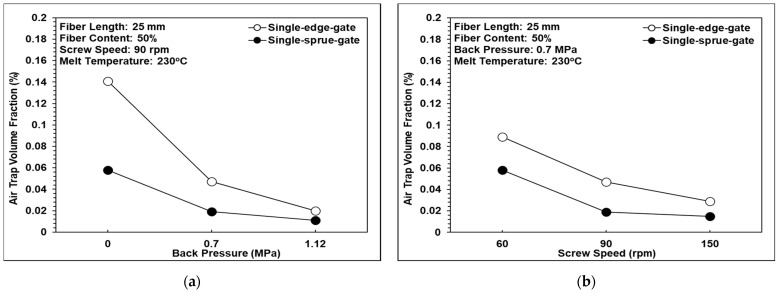

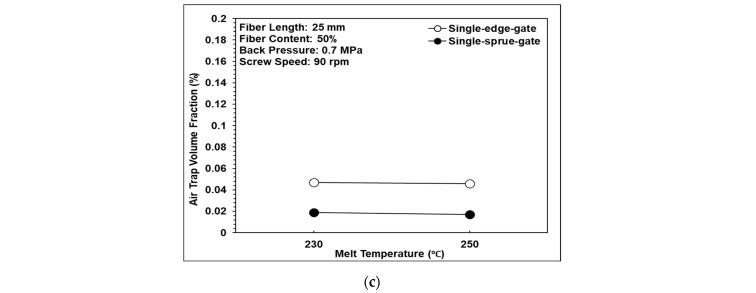
Effects of the different melt flow paths on the air trap volume fraction: (**a**) back pressure variations, (**b**) screw speed variations, and (**c**) melt temperature variations.

The existence of air traps in fiber-reinforced composites significantly reduces their mechanical performance. Figure 15 shows the effects of back pressure, screw speed, and melt temperature, respectively, on the air trap volume fraction in the present samples. It can be observed that as the back pressure and the screw speed were increased, the volume fraction of the air traps reduced due to the corresponding increase in the pressure gradient, which expels the trapped air within the composite resin. However, the melt temperature had no obvious effect on the air trap volume fraction. The air trap volume fraction within the single-edge-gate samples was higher than that in the single-sprue-gate samples. This finding is reasonable since the flow path in the single-edge-gate cavity was longer than that in the single-sprue-gate cavity and, hence, the pressure gradient and the shear strain rate were smaller. Consequently, the trapped air was more difficult to expel.

### 3.7. Tensile Strength Analysis

**Figure 16:**
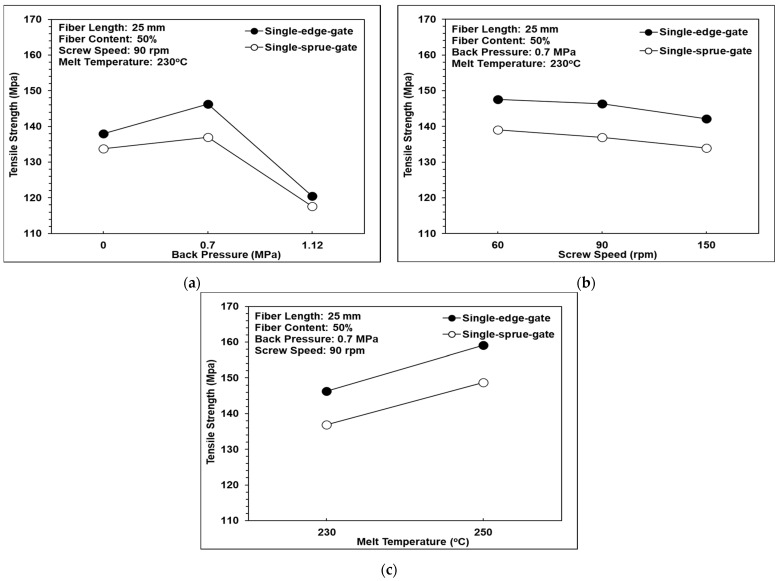
Effects of the different melt flow paths on tensile strength: (**a**) back pressure variations, (**b**) screw speed variations, and (**c**) melt temperature variations.

Figure 16a shows that as the back pressure was increased from 0 to 0.7 MPa, the tensile strength also increased. However, as the back pressure was further increased, to 1.12 MPa, the tensile strength decreased. This tendency is reasonable since for back pressure values of 0-0.7 MPa, the fiber length remained approximately constant, at around 10 mm (see Figure 10a), while the volume fraction of the air traps reduced (see Figure 15a). However, at higher back pressure, the fiber length reduced dramatically, to around 5 mm, and hence, the tensile strength was reduced significantly despite the further reduction in the air trap volume fraction. In addition, the tensile strength of the single-edge-gate specimens was higher than that of the single-sprue-gate specimens due to relatively longer fiber length in the single-edge-gate samples, as discussed in previous sections.

Figure 16b shows that as the screw speed was increased from 60 rpm to 150 rpm, the tensile strength was reduced. In Figure 10b and Figure 15b, it can be observed that the rate of reduction in the volume fraction of the air traps with increases in the screw speed was greater than that of the fiber length. In the case of composite materials, a lower volume fraction of air traps increases the tensile strength, while a shorter fiber length reduces the tensile strength. Consequently, the results shown in Figure 16b suggest that in the long-fiber-reinforced thermoplastic parts investigated in this work, the tensile strength was dominated by the fiber length rather than the volume fraction of air traps.

Figure 16c shows that as the melt temperature was increased from 230 °C to 250 °C, the tensile strength also increased. The results presented above show that the melt temperature has no significant effect on the fiber length, fiber orientation, fiber weight fraction, or volume fraction of air traps. Hence, it can be inferred that the improved tensile strength of the long-fiber-reinforced thermoplastic parts at higher melt temperatures is the result of the greater fluidity and better bonding of the composite melt, which leads to a more uniform dispersion of the fibers within the matrix.

## 4. Conclusions

In this study, the effects of back pressure, screw speed, and melt temperature on the fiber characteristics and mechanical properties of long-fiber-reinforced thermoplastic parts were examined using a customized injection molding machine with a three-barrel injection system. The experiment results support the following significant findings and conclusions:

1. The experiments used 25 mm fiber length, and the results show that using the three-barrel injection molding machine, the fibers in the molded components retained a maximum average length of around 10 mm, which was much longer than that of the fibers in the parts produced using a traditional injection molding machine [8,20]. In other words, the effectiveness of the three-barrel-type injection unit in terms of minimizing the breakage of the reinforcing fibers is confirmed.
2. The fiber length results show that fiber breakage was dominated by the back pressure and the screw speed. By increasing the back pressure and the screw speed, the fiber length can be decreased to 57% and 37%, respectively. In contrast, the melt temperature had only a minor effect on the fiber length.
3. In fiber orientation results, it was found that most of the fibers within the long-fiber-reinforced thermoplastic parts were aligned in the flow direction due to the shallow geometry of the molded part (i.e., a tensile testing specimen) and the inherent long nature of the fibers. The back pressure, the screw speed, and the melt temperature do not significantly affect the fiber orientation because the fiber length is considerably long.
4. The air trap volume fraction results show that the volume fraction of the air traps in the single-edge-gate samples was higher than that in the single-sprue-gate samples due to the longer melt flow distance, which reduced both the pressure gradient and the strain rate during the molding process. In the case of both sample types, a higher back pressure and screw speed increased the pressure gradient and, therefore, reduced the volume fraction of the air traps. For example, increasing the back pressure from 0 to 1.12 MPa reduced the air trap volume fraction by 86% while increasing the screw speed from 60 to 150 rpm reduced the air trap volume fraction by 67%.
5. The tensile strength results show that as the back pressure was increased from 0 to 0.7 MPa, the fiber length was maintained and the volume fraction of the air traps was reduced. Consequently, the tensile strength of the molded components increased. However, as the back pressure was further increased, to 1.12 MPa, severe breakage of the fibers occurred. Hence, the tensile strength decreased. As the screw speed was increased from 60 to 150 rpm, the tensile strength also decreased since the dominant factor was the fiber length, even when the volume fraction of the air traps declined. The melt temperature had no significant effect on the fiber length, the fiber orientation, or the volume fraction of the air traps. However, as the melt temperature was increased, the fluidity of the melt increased and, hence, the fibers were dispersed more uniformly throughout the matrix. Consequently, the tensile strength increased.

## Figures and Tables

**Table 1:** Configuration of the injection-molded long-glass-fiber-reinforced polypropylene composites.

A. Parameters					
Controllable Factors				Factors Maintained Constant	
Back pressure (MPa)	0	0.7	1.12	Injection pressure (%)	70
Screw speed (rpm)	60	90	150	Injection speed (mm/s)	60
Melt temperature (°C)	230	250		Injection time (s)	2
				Packing pressure (%)	30
				Packing time (s)	10
				Mold temperature (°C)	80
				Cooling time (s)	30
**B. The treatments used to mold the samples in random order**					
**Experiment**	**Back pressure (MPa)**			**Screw speed (rpm)**	**Melt temperature (°C)**
1	0			90	230
2	0.7			90	230
3	1.12			90	230
4	0.7			60	230
5	0.7			150	230
6	0.7			90	250

**Table 2:** Computerized tomography parameters.

Parameter	Value
Voltage	130
Current (mA)	120
Resolution (pixels)	1536 × 1920
Pixel size (mm)	0.127
Voxel size (μm)	42

## Data Availability

The data presented in this study are available on request from the corresponding author.
